# Opportunities and challenges for Genomic Data Analyses in Biobanks: a call for papers

**DOI:** 10.1093/genetics/iyae023

**Published:** 2024-03-04

The journals of the Genetics Society of America, GENETICS and G3: Genes|Genomes|Genetics, are calling for submissions of papers on biobank-scale genomic data analyses.

Over the past decade, biobanks—defined as large collections of biological, medical, and genetic data on the same individuals—have revolutionized human genomics research by deepening our understanding of the complex relationships between genomes and phenomes at different organizational levels (e.g. tissues, individuals, and populations). Although the biobank model is becoming the new standard for data collection, substantial heterogeneity subsists between biobanks across the world creates both new challenges and new opportunities for data analysis.

This series will highlight theoretical, analytical, and computational advances addressing these challenges and leveraging these opportunities.

Specifically, we invite high-quality submissions with a focus on broad- and fine-scale population structures (caused by demography, evolutionary forces or ascertainment) in biobanks; the effect of selection bias and measurement errors on statistical methods to detect genetic associations (and interactions); clinically informative observations in medical records (particularly in hospital-based biobanks); linkage with external data sources (for example, to map social determinants of health); challenges with meta-analysis across biobanks; computationally-scalable analysis methods (e.g. involving large numbers of individuals and traits); family-based analyses; multi-traits analyses; causal inference; longitudinal electronic health record data analysis; and genomic prediction of health-related and social traits.

In addition, this series welcomes submissions addressing ethical and privacy-related issues in data analysis, interfaces for disseminating results (e.g. phenome-wide association studies browsers), and technological considerations for omics data generation (e.g. SNP-array genotyping vs whole-exome or whole-genome sequencing).

**Series Editors:**

- Mattias Jakobsson, Uppsala University, Sweden
- Bhramar Mukherjee, University of Michigan, United States of America
- Loic Yengo, University of Queensland, Australia
- Noah Zaitlen, University of California, Los Angeles, United States of America

The Series will launch in early 2025, though articles will be published online shortly after acceptance via Advance Access. Papers will be collected and navigable across the two journals and promoted on social media and other venues in a manner similar to other series the journals have published.

Authors are invited to submit manuscripts by 2024 May 31, and should submit to their journal of choice. Manuscripts will be reviewed and edited through the standard peer review process and according to the usual high standards of the journals. Some manuscripts submitted to GENETICS may be offered a transfer to G3: Genes|Genomes|Genetics instead.

We encourage continued submissions past 2024 May 31 as we intend this series to be an ongoing and growing resource in this area. At submission, please choose the “Genomic Data Analyses in Biobanks” article type in the submission system and mention the series in your cover letter.

Please share with your colleagues, and do not hesitate to contact any of the series editors or the GENETICS and G3: Genes|Genomes|Genetics Editorial Office with questions, suggestions, or presubmission inquiries.

